# Optical Genome Mapping Reveals Frequent Cryptic Structural Aberrations in Normal Karyotype Acute Myeloid Leukemia

**DOI:** 10.1002/ijc.70548

**Published:** 2026-05-14

**Authors:** Tuuni Turtinen, Andriana Valkama, Christopher Wray, Sandra Vorimo, Hannele Räsänen, Eeva‐Riitta Savolainen, Katri Pylkäs, Tuomo Mantere

**Affiliations:** ^1^ Laboratory of Cancer Genetics and Tumor Biology, Translational Medicine Research Unit, Medical Research Center Oulu and Biocenter Oulu University of Oulu Oulu Finland; ^2^ Northern Finland Laboratory Centre Nordlab Oulu Finland

**Keywords:** acute myeloid leukemia, cytogenetics, normal karyotype, optical genome mapping, structural variants

## Abstract

Approximately half of newly diagnosed acute myeloid leukemia (AML) cases are cytogenetically normal (CN) when analyzed with conventional karyotyping. However, CN‐AML exhibits a wide range of clinical heterogeneity, which may partly be explained by structural variants (SVs) that are not detected with current standard cytogenetic techniques. Here, 48 CN‐AML cases were analyzed using optical genome mapping (OGM) for comprehensive SV assessment and to identify novel candidate gene alterations. Abnormalities were detected in 22 of 48 cases (46%). Large SVs, or those affecting leukemia‐associated genes, were identified in 16 cases (33%), encompassing 18 abnormalities. Copy‐neutral loss‐of‐heterozygosity regions were detected in seven cases (15%), and they were mutually exclusive with the presence of SVs in all but one case. SVs included eight deletions, six partial tandem duplications, two balanced translocations, and two complex rearrangements. The most frequently altered genes were *KMT2A* (5 cases) and *RUNX1* (3 cases), followed by deletions of *NF1* and the 13q14 (*DLEU*) region (2 cases each). Single alterations included *NUP98::NSD1* and deletions of *TET2*, *PRPF8*, and *FLT3*. In addition, as a novel finding, we identified a balanced translocation t(3;20)(p13;q13.12) leading to a putative *FOXP1::EYA2* fusion. Notably, the presence of OGM‐detected abnormalities was associated with worse disease‐specific survival (Mantel–Cox test, *p* = 0.007). Overall, this study demonstrates that a significant proportion of CN‐AML cases harbor clinically relevant SVs, especially those associated with adverse prognosis, that escape detection by standard techniques. Our results support the use of OGM as a streamlined, genome‐wide tool for both research and diagnostic applications in AML.

## Abstract (highlights)

What's new?

Acute myeloid leukemia adult cases often appear cytogenetically normal when analyzed with conventional karyotyping. However, acquired structural variants may escape routine detection. Here, optical genome mapping detected diverse genomic alterations in nearly half of the analyzed cytogenetically normal cases. These included recurrent alterations of *KMT2A*, *RUNX1*, *NF1*, and *DLEU* as well as *NUP98::NSD1* and a putative novel *FOXP1::EYA2* fusion. The presence of abnormalities was associated with worse survival. The results underscore optical genome mapping as a powerful genome‐wide tool for detecting all types of structural alterations and for improving the understanding of clinical heterogeneity in cytogenetically normal acute myeloid leukemia.

## Graphical abstract

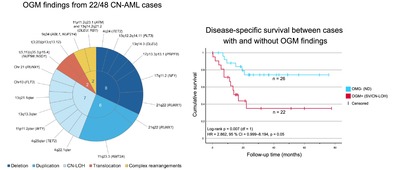

**Abbreviations**
AML: acute myeloid leukemia
CN: cytogenetically normal
CN‐LOH: copy‐neutral loss‐of‐heterozygosity
CNV: copy number variant
DNP: de novo pipeline
DSS: disease specific survival
ELN: European LeukemiaNet
FHRB: the Finnish Hematology Registry and Clinical Biopank
HR: hazard ratio
InDel: insertion–deletion
ITD: internal tandem duplication
MAF: minor allele frequency
NGS: next‐generation sequencing
OGM: optical genome mapping
OS: overall survival
PTD: partial tandem duplication
SNP: single nucleotide polymorphism
SNV: single nucleotide variant
SV: structural variant
VAF: variant allele frequency
WHO: World Health Organization

## 1 Introduction

Acute myeloid leukemia (AML) is a heterogeneous malignancy of the hematopoietic system characterized by abnormal proliferation of immature myeloid cells [1]. This abnormal growth leads to the accumulation of blasts within the bone marrow and causes pancytopenia. The annual age‐adjusted incidence rate of AML is around 4.3 cases per 100,000 people, and the current overall 5‐year relative survival rate in adult AML patients remains only around 30% in Western populations [2, 3].

Recurrent chromosomal abnormalities and structural variants (SVs) have served as important diagnostic and prognostic markers in AML for decades and provided critical insights into disease pathomechanisms [4]. However, in about half of the adult AML cases, conventional karyotyping does not reveal abnormalities, making cytogenetically normal AML (CN‐AML) the most common cytogenetic subgroup of AML. Patients with CN‐AML are generally classified into favorable and intermediate risk categories according to the European LeukemiaNet (ELN) classification system, in the absence of adverse‐risk molecular mutations [5]. However, there is a considerable degree of heterogeneity within this group in terms of clinical outcomes, which poses prognostic and therapeutic challenges, for example, when considering indications for stem cell transplantation [1].

Beyond chromosomal alterations, advancements in sequencing technologies have led to the discovery of various genes affected by single nucleotide variants (SNVs) and small insertions and deletions (InDels) in AML [6]. These findings are widely integrated into the diagnostic workflows and have advanced prognostic evaluations. In CN‐AML, sequencing approaches detect mutations in at least one well‐established leukemia gene in approximately 98% of cases [7]. However, the genome‐wide prevalence of acquired SVs that may escape detection by routine karyotyping remains unclear in CN‐AML, warranting further investigations with high‐resolution techniques. Accurate detection and characterization of these hidden SVs may reveal novel candidate genes involved in AML pathogenesis and provide clinically relevant information. To date, few studies have applied the latest genomic techniques to analyze CN‐AML. Here, we used optical genome mapping (OGM) to explore the prevalence and types of hidden SVs in this group. Previous studies have shown that OGM enables genome‐wide detection of both balanced and unbalanced large‐scale SVs at high resolution and can identify SVs at variant allele frequencies (VAFs) as low as 5%–15% [8, 9, 10, 11, 12, 13, 14, 15, 16, 17]. These features, along with cost‐effectiveness and a low number of false positive SV calls [18], make it an efficient tool for elucidating the hidden SV landscape of CN‐AML.

## 2 Materials and Methods

### 2.1 Samples

This study encompassed 48 diagnostic stage samples from adult AML patients (aged 18–84 at diagnosis; 52% females) diagnosed between 2018 and 2024. All cases had undergone routine diagnostic karyotyping (Giemsa staining) and gene panel sequencing analysis and presented a normal karyotype (46, XX or 46, XY), analyzed according to current recommendations, requiring at least 20 metaphases. Any additional routine fluorescence in situ hybridization (FISH) testing targeting 3q26 (*MECOM*), 5q31 (*EGR1*), 7q31, 11q23 (*KMT2A*), and 17p13 (*TP53*) were also negative. Samples used in this study were mononuclear cell pellets derived from bone marrow (stored at −80°C) and they were provided by The Finnish Hematology Registry and Clinical Biobank (FHRB) (Vantaa, Finland: http://www.fhrb.fi). The disease‐related information obtained from FHRB included WHO [4], ELN [5], and FAB [19] classifications, mutations from gene panel next‐generation sequencing (NGS) performed by diagnostic laboratories, age at diagnosis, sex, follow‐up time, alive/deceased status and cause of death (Table S1). The diagnostic NGS results were based on two gene panels that largely share the same genes. Details of gene content, minimum read depth, and sequencing technology are presented in Table S2.

### 2.2 DNA Extraction, Labelling and Data Collection for OGM

Ultra‐high molecular weight (UHMW) genomic DNA (gDNA) was extracted from frozen mononuclear cell pellets using Bionano Prep Sp‐G2 Blood and Cell DNA Isolation Kit according to the Bionano Prep SP‐G2 Frozen Cell Pellet DNA Isolation Protocol and quantified with Qubit Fluorometer 3.0 (Qubit BR dsDNA assay kit; ThermoFisher Scientific). The extracted gDNA (750 ng) was labelled using DLE‐1 enzyme (fluorescent tags) with the Direct Label and Stain (DLS) technique (Bionano Prep DLS‐G2 Labeling Kit) according to the manufacturer's instructions (Bionano Genomics), followed by Qubit Fluorometer quantification (Qubit HS dsDNA assay kit, ThermoFisher Scientific). After determining the final concentration of gDNA, the labelled and stained samples were loaded on Saphyr chips (G3.3) and run on the Saphyr instrument (Bionano Genomics) for visual imaging. For each sample, the data collection was preset to 1800 Gbp, and hg38 was used as the reference genome.

### 2.3 OGM Data Analysis

OGM analysis was conducted in two steps. The first step followed a diagnostic‐type cytogenetic analysis, focusing on large alterations (> 5 Mbp) and those overlapping clinically relevant and/or leukemia‐associated genes (Table S3). The second step was discovery‐oriented, aiming to identify novel candidate genes by focusing on recurrently altered genes and putative novel fusion genes in the cohort. For non‐recurrent smaller SVs (< 5 Mb) that did not overlap with known leukemia‐associated genes, a VAF below 20% was used as the threshold for likely acquired alterations. The Rare Variant Pipeline v3.8 (RVP) was used for SV and copy number variant (CNV) detection, and the *De Novo* Pipeline v3.8 (DNP) was applied to identify large copy‐neutral loss‐of‐heterozygosity (CN‐LOH) regions spanning telomeres. Alterations were visualized with Bionano Access software (v1.8), and manual curation of inter‐ and intrachromosomal rearrangements was performed according to the guidelines provided in Levy et al. [18]. Default masking and recommended confidence scores were applied for SV and CNV detection (insertion: 0, deletion: 0, inversion: 0.7, duplication: −1, intra‐translocation: 0.02, inter‐translocation: 0.02, copy number: 0.99, and aneuploidy: 0.95). Recommended size cutoffs of 500 kbp and 25 Mbp were applied for CNV and CN‐LOH calling, respectively. All SVs present in the OGM population control database of 285 individuals provided by Bionano Genomics, as well as those found in our in‐house control cohort of 109 Finnish individuals, were filtered out.

### 2.4 Confirmation of CN‐LOH Events Utilizing SNPs Co‐Occurring With Label Sites

To evaluate the reliability of CN‐LOH calls from the DNP, we applied an additional alternative approach: if a nucleotide within the ‘CTTAAG’ label site is altered, the enzyme cannot recognize the motif, resulting in a missing label at the corresponding reference position in the assembly. The gnomAD database (v.4.1.0) was used to retrieve all single nucleotide polymorphisms (SNPs) co‐occurring with label sites in the OGM reference genome (hg38_DLE1_0kb_0labels_masked_YPARs.cmap). Only SNPs with a total minor allele frequency (MAF) > 0.05 in GnomAD were included in the analysis. Label sites with overlapping SNPs were investigated using the assembly data (exp_refineFinal1_merged_r.cmap) from CN‐LOH regions of positive cases (AML3, AML33, AML39, AML43, AML44, AML47, and AML48) and from a control case without CN‐LOH (AML22). The presence or absence of these labels in the assemblies was then used to indirectly determine the allelic states of the co‐occurring SNPs.

### 2.5 RNA Sequencing and Data Analysis

The total RNA was extracted using RNeasy plus Mini Kit (Qiagen) from five cases (AML15, AML28, AML29, AML30 and AML47) with suitable residual material available. A total of 1000 ng RNA was used for the TruSeq Stranded mRNA Library Preparation (Illumina) following the manufacturer's instructions. Quantification and quality assessment of the libraries were performed using Bioanalyzer 2100 with High Sensitivity DNA Kit (Agilent), Qubit Broad Range DNA‐kit (ThermoFisher Scientific) and qPCR NEBNext Library Quant Kit (NEB). The RIN‐values for the RNA samples ranged from 6.4 to 9.5. Libraries were sequenced using Illumina NextSeq550 platform in high‐output, pair‐ended 2 × 76 cycle mode, followed by FASTQ generation. The resulting mRNA‐Seq data was analyzed with DRAGEN RNA pipeline (v.4.4.4001) enabling gene expression quantification and fusion gene detection using GRCh38 (hg38) as the reference genome. Subsequent differential gene expression analysis was performed using DRAGEN Differential Expression analysis (v.4.3.7), comparing the three *KMT2A* partial tandem duplication (PTD) cases with two non‐*KMT2A*‐PTD cases. For cases with *KMT2A‐*PTD, the Integrative Genomics viewer (IGV) v.2.16.1 was used to manually evaluate the aligned reads to characterize the exonic configuration of the PTD. The sequencing coverage and quality statistics for each sample are summarized in Table S4.

### 2.6 Statistical Tests

Kaplan–Meier survival analysis (log‐rank Mantel‐Cox test) and Cox proportional hazards model with age as a covariate were used to compare survival and hazard ratios (HRs) between cases with and without OGM findings (defined as SVs > 5 Mb, SVs overlapping with leukemia‐associated genes and CN‐LOHs). Disease‐specific survival (DSS) was analyzed using AML‐related death as the event. Follow‐up time was measured in months from diagnosis to either the event or the last follow‐up. Associations between OGM status (positive/negative) and categorical variables, such as the ELN2022 risk category and *NPM1* mutation status, were assessed using a two‐sided *χ* ^2^ test. In analyses involving ELN2022 groups, the intermediate‐ and adverse‐risk subgroups were combined into a “non‐favorable” group due to the small number of adverse‐risk cases. The *χ* ^2^ test was also used to compare the proportion of heterozygous SNP label sites between the genomic regions of CN‐LOH positive cases and a control sample. All statistical analyses were performed using IBM SPSS Statistics for Windows, version 29.0.1.1 (IBM Corp., Armonk, NY, USA), and *p*‐values < 0.05 were considered statistically significant.

## 3 Results

### 3.1 OGM Quality Metrics

The OGM preparation and processing were successful for all the 48 samples. The total amount of molecule data collected was on average 1654 Gbp (min: 476, max: 1877) per sample and an effective coverage per sample was on average 462× (min: 106, max: 558). Map rates were above the recommended 70% for 45/48 samples; three of the samples had map rates below the recommended range (minimum: 63%). The number of labels per 100 kbp was on average 15.7 (min: 14.0, max: 17.4, recommended range 14–17). The summaries of OGM technical quality metrics are presented in Table S5.

### 3.2 Overall OGM Results Combined With NGS Results

**FIGURE 1:**
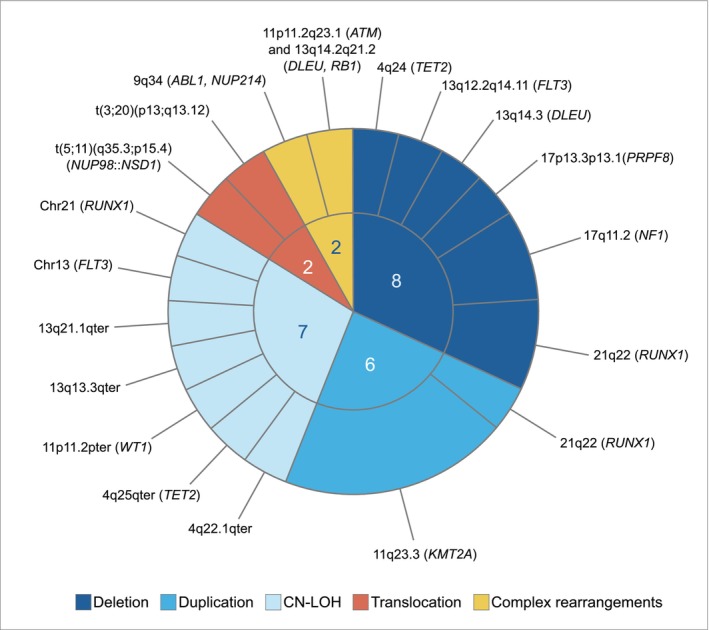
OGM findings in 22 out of 48 CN‐AML cases. The affected leukemia‐associated genes are shown in parentheses for structural variants (deletions, duplications, translocations, and complex rearrangements). For CN‐LOH events, the gene shown in parentheses carries an SNV/InDel detected by gene panel sequencing analysis within the CN‐LOH region.

OGM analysis revealed a total of 1271 rare SVs and 65 CNV segments in the cohort. Overall, the mean number of rare SV calls per case was 26. Aneuploidies were not detected and the mean for the number of CNV segment calls was 1.35 (range: 0–15, median: 0). Out of these, 18 of these alterations were categorized as clinically relevant or reportable findings (either due to their large size above 5 Mbp or involvement of known leukemia‐associated genes) and were present in 16 out of 48 (33%) cases. These included eight deletions, six intragenic partial tandem duplications (PTDs), two balanced translocations, and two complex rearrangements. In addition, seven large (minimum size 44 Mbp) CN‐LOH regions entailing telomeric regions were identified. When combining CN‐LOH and SVs, alterations were present in 22 out of 48 cases (46%) (Figure 1 and Table S1). The most common reason for the alterations to be missed previously was the small size (60%), followed by CN‐LOH (28%), which is undetectable by karyotyping. In addition, one of the identified translocations (leading to *NUP98::NSD1* fusion) is a known cryptic rearrangement. For two of the alterations (reciprocal translocation and a complex rearrangement), the reason they were missed remains unclear and cannot be unambiguously explained by the size of the SVs. The most likely reasons why the alterations were missed previously are summarized in Table S1.

**FIGURE 2:**
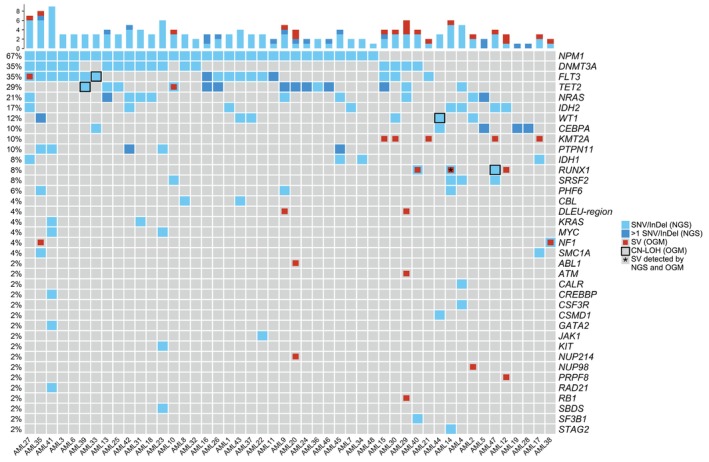
Overview of altered leukemia‐associated genes combined from OGM analysis and diagnostic gene panel sequencing. Cases are presented in columns and altered genes in rows. The percentages of cases with alterations in a given gene are shown on the left. The bars on top indicate the number of SNVs/InDels/SVs in each individual case. A total of 166 mutations were identified by NGS, of which 131 were single‐hit mutations. SVs affecting leukemia‐associated genes were present in 16 cases. There were four cases in which CN‐LOH encompassed a gene that also had a mutation detected by NGS (black borders). Asterisk (*) in *RUNX1* refers to an SV which was detected by both NGS and OGM. OncoPrint image was created in R with ComplexHeatmap package [20].

For a comprehensive overview of altered genes in CN‐AML, OGM results were combined with diagnostic panel sequencing results received from FHRB. In total, diagnostic panel sequencing had revealed 166 mutations across 29 different genes, with an average of 3.4 mutations per sample (range 1–9). All altered leukemia‐associated genes identified in the studied CN‐AML cohort, including both SVs and SNVs/InDels, are presented in figure 2 prepared with ComplexHeatmap package [20]. In eight cases, the combination of OGM and NGS confirmed biallelic or multi‐hit mutational status of a leukemia‐associated gene by detecting a deletion or CN‐LOH co‐occurring with a pathogenic SNV/InDel. This included deletions of *TET2* (AML10), *FLT3* (AML27), *NF1* (AML38) and *RUNX1* (AML40); and CN‐LOH regions entailing *FLT3* (AML33), *TET2* (AML39), *WT1* (AML44), and *RUNX1* (AML47). Of note, 76% of all genes with alterations (detected by OGM or NGS) are included in both NGS panels (Table S2), while 16% (*ATM, CSMD1, KMT2A, MYC, RB1* and *SBDS*) are present only in gene panel 1. The remaining 8%, comprising *NUP98*, *NUP214*, and the *DLEU* region, with SVs detected by OGM, are not included in either NGS panel.

### 3.3 Gene‐Level Information and Characterization of the OGM Identified SVs

Additional findings from OGM analysis entailed various types of both recurring and individual alterations, which were categorized by mutation types and altered genes. The most frequent additional finding in the cohort was the *KMT2A*‐PTD observed in five (AML15, AML17, AML21, AML30 and AML47) out of 48 cases (10%). Due to their small size, these were called interstitial insertions by OGM. All the insertions showed a repeat of the same region entailing three‐labels and were visually interpreted as PTDs (Figure S1). For three cases (AML15, AML30 and AML47) we had suitable material left for RNAseq analysis, which confirmed that the PTD entailed exons 2 to 8 corresponding to the canonical and most frequent form of *KMT2A*‐PTD. We also compared the most differentially up‐ and downregulated genes in the three *KMT2A*‐PTD cases to those in two control cases without the alteration. This showed a significant differential expression of several genes (Table S6), but no clear enrichment of genes in specific pathways was identified.

Following *KMT2A*‐PTD, and as the second most common gene affected by SVs, *RUNX1* disruptions were detected in three separate cases by OGM (Figure S2). Two of these involved heterozygous deletions, one being a relatively small 2.5 kbp deletion (VAF: 23%) entailing exons 1 and 2 of *RUNX1* (AML12) detected by the DNP. The other was a 1.9 Mbp deletion (VAF: 45%) covering the entire gene (AML40), which together with a point mutation detected in NGS indicated biallelically mutated *RUNX1*. In addition, one case (AML14) was identified with an intragenic *RUNX1* PTD entailing exons 3 to 6 (VAF: 19%), which had also been detected with NGS‐based CNV‐analysis. For AML12 and AML14, the VAFs of the *RUNX1* SVs were < 25%, strongly indicating a somatic origin, whereas for the other *RUNX1* alterations (including SNVs), we cannot rule out a germline origin based on the VAFs. Other recurrent alterations included deletions of the *DLEU* (13q14) region and *NF1*. In two cases, OGM revealed deletions encompassing the *DLEU* region: a 1.5 Mb deletion at 13q14.3 and a 12.2 Mb deletion at 13q14.2q21.2 (AML9 and AML29, respectively) (Figures S3A and S4A). The latter formed a part of a complex rearrangement that also resulted in deletions of *RB1* (13q) and *ATM* (11q). Both alterations in *NF1* were simple deletions. One was a 1.3 Mbp deletion spanning the entire gene (AML35), while the other was a 50 kbp deletion predicted to remove exons 36–56 (AML38) (Figure S3B,C). The latter case also harbored a small frameshift mutation in *NF1* exon 18 detected by NGS (VAFs: 34% for the frameshift, 41% for the deletion), indicating a likely biallelic loss of *NF1*.

In addition, simple deletions involving leukemia‐associated genes were identified in *TET2*, *PRPF8*, and *FLT3* (Figure S3D–F) A 430 kbp deletion of *TET2* (AML10) encompassed the entire gene and co‐occurred with a stop‐gain mutation in *TET2* with a high VAF of 88%, establishing biallelic *TET2* mutation status. In AML12, visual inspection revealed a deletion spanning the whole *PRPF8* gene, which was initially classified as an intrachromosomal fusion by the SV algorithm due to its large size (5.6 Mbp). Similarly, a 12.8 Mbp deletion of 13q12.2q14.11 (AML27) was identified and predicted to include exons 1–9 of *FLT3*. This deletion co‐occurred with an internal tandem duplication (ITD) of *FLT3* exon 14 detected by NGS.

Two cases in the studied cohort harbored complex rearrangements: one comprising connected inter‐ and intrachromosomal rearrangements affecting chromosomes 11 (p11.2–q23.1) and 13 (q14.2–q21.2), leading to deletions of *ATM*, *RB1*, and the *DLEU* region (AML29) (Figure S4A) and another one involving multiple SVs in the 9q34 region overlapping *ABL1* and *NUP214* (AML20) (Figure S4B). Importantly, OGM also identified a t(5;11)(q35.3;p15.4) translocation resulting in the *NUP98::NSD1* fusion gene in AML2, which was the sole known fusion gene detected in the studied cohort (Figure S5A).

### 3.4 Genes Outside the Set of Known Leukemia‐Associated Genes

Smaller SVs and those that did not overlap with the leukemia‐gene list (Table S3) comprised a total of eight events. One of these was a reciprocal balanced translocation, t(3;20)(p13;q13.12), which has not been previously reported in the literature. Interestingly, this balanced translocation could potentially generate a fusion between the transcription factor *FOXP1* and the transcriptional coactivator and phosphatase *EYA2* (Figure S5B). However, RNA extracted from this case was too degraded for RNA‐seq, and attempts using cDNA‐based sequencing also failed to confirm the presence of a fusion transcript. In addition, the smaller somatic SVs without overlap with the leukemia‐gene list included five deletions (11.7 kb–2.7 Mb), an interchromosomal insertion from chromosome 2 into chromosome 22, and an inversion on chromosome 1 (affected genes listed in Table S7).

### 3.5 Copy‐Neutral Loss‐of‐Heterozygosity

Large CN‐LOH regions involving telomeres were identified in 7 out of 48 (15%) cases. These included chromosomal regions 4qter (2 cases), 11pter (1 case), 13qter (2 cases), and whole chromosomes 13 (1 case) and 21 (1 case) (Figure 1). The sizes of the CN‐LOH regions ranged from 45 to 114 Mb. In four out of seven CN‐LOH regions, a mutation with a high VAF (ranging from 72% to 96%) was detected by NGS. These included a *WT1* (11p) stop‐gain mutation (AML44), a *RUNX1* (21q) missense mutation (AML47), an *FLT3*‐ITD (13q) (AML33), and a *TET2* frameshift mutation (4q) (AML39) (Figure 2, Table S1). In addition to the detection of pathogenic mutations with high VAFs in CN‐LOH regions, an orthogonal approach was applied to assess the allelic states of common polymorphisms co‐occurring with OGM label sites (see Materials and Methods). Here, the reference genome used for OGM analysis contained 642,985 label sites, covering approximately 3.86 Mbp of the genome. SNP frequencies and genomic positions retrieved from gnomAD revealed 7809 SNPs with a MAF > 0.05 overlapping these sites in the autosomes. Within CN‐LOH regions defined by the DNP in the studied cohort, the number of label sites ranged from 6600 to 22,000, with 86 to 299 co‐occurring with SNPs per LOH region. Heterozygosity at these informative label sites was significantly reduced in cases with CN‐LOH calls when compared with the corresponding regions in a control sample (*p* < 0.001), ranging from 0% to 7.6% (mean: 2.0%) in CN‐LOH–positive cases versus 20.5%–24.1% in the control sample (Table S8).

### 3.6 OGM Finding Associates With Lower Survival in the Cohort

**FIGURE 3:**
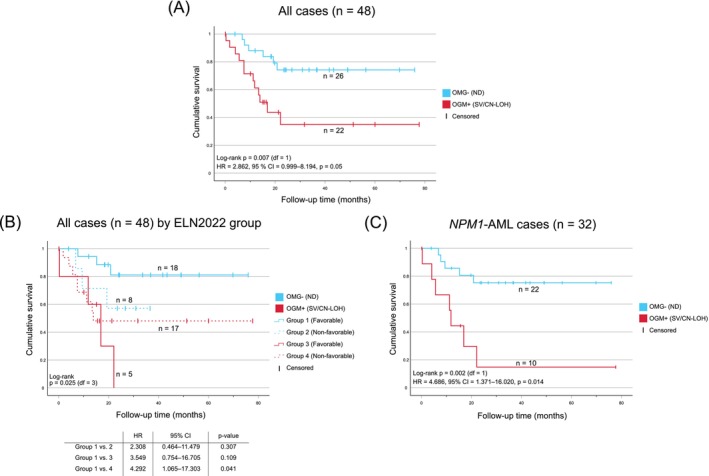
Kaplan–Meier curves showing cumulative survival and age‐adjusted hazard ratios for cases with an SV/CN‐LOH detected by OGM (red) and cases without significant OGM findings (blue) (ND: Not detected). Cases were right‐censored at the time of the latest follow‐up. (A) Disease‐specific survival of all cases. (B) Disease‐specific survival of all cases stratified by ELN2022 risk classification. (C) Disease‐specific survival of *NPM1*‐mutated cases.

In the studied cohort, 38% (*n* = 18) of patients had died from AML and 6% (*n* = 3) from other causes. When comparing survival between cases with an OGM finding (defined as SVs > 5 Mb and/or overlapping with a leukemia‐associated gene and/or CN‐LOH) and those without, survival was worse in the group with OGM findings (*p* = 0.005 for OS and *p* = 0.007 for DSS, Kaplan–Meier analysis, log‐rank Mantel–Cox test) (Figure 3A). This group, representing 22 cases (46%), accounted for 14 of 21 (67%) of all deaths and 12 of 18 (67%) of AML‐related deaths. According to an age‐adjusted Cox proportional hazards model, the risk of AML‐related death was higher among patients with an OGM finding (HR = 2.862), although the result was only borderline significant (95% CI = 0.999–8.194, *p* = 0.05). The mean age of patients with an OGM finding was 66 years (range: 30–84), compared with 62 years (range: 18–83) in those without OGM findings.

Following this observation, we assessed the association between OGM status and ELN2022 risk classification categories. OGM‐positive cases were associated with non‐favorable risk categories (OR = 7.65, 95% CI = 2.09–28.05, *p* = 0.002), accounting for 17/25 (68%) of the non‐favorable cases. Here, a borderline significant difference in survival was observed between OGM‐positive cases of non‐favorable risk category and OGM‐negative cases of favorable category (HR = 4.29, 95% CI = 1.07–17.30, *p* = 0.041). Notably, also the OGM‐positive cases within the favorable ELN risk category showed poor survival, although the small number of patients in this group (*n* = 5) is limiting the statistical power of the analysis (Figure 3B). In line with the association with non‐favorable risk category, the OGM‐positive cases were more likely to be *NPM1*‐negative (OR = 6.6, 95% CI = 1.7–25.62, *p* = 0.006). Interestingly, within the *NPM1*‐mutated group (*n* = 32), survival was worse in the OGM‐positive group (log‐rank *p* = 0.002), with an HR of 4.69 (95% CI = 1.37–16.02, *p* = 0.014) (Figure 3C).

## 4 Discussion

CN‐AML represents the largest cytogenetic subgroup in adult AML, accounting for 40%–50% of newly diagnosed cases. It is a clinically heterogeneous group, most often classified into intermediate‐ or favorable‐risk categories depending on additional molecular genetic findings [1]. While standard karyotyping remains a crucial tool in AML diagnostics, it lacks the resolution to detect submicroscopic SVs and various cryptic rearrangements, as well as to characterize visible alterations down to the gene level [21]. OGM, in contrast, allows simultaneous genome‐wide detection of all major SV classes in a single assay, providing an unprecedented opportunity to interrogate the complete SV landscape in CN‐AML. In this study, we applied OGM analysis to comprehensively assess SVs in 48 CN‐AML cases and to evaluate the frequency, types, and potential clinical relevance of the detected alterations.

Altogether, OGM revealed SVs (including large events and those involving leukemia‐associated genes) or CN‐LOH events in nearly half of the cases (46%). The types included different sizes of deletions, intragenic duplications, balanced translocations, complex rearrangements, and CN‐LOH regions. Most additional alterations detected by OGM were missed by karyotyping due to the small size of the SVs. However, other reasons, including LOH and a cryptic translocation, were also noted. Two of the alterations, in principle, could have been detected by karyotyping, and the reason they were missed remains unclear. Our findings confirm that CN‐AML is far from ‘silent’ with respect to its SV landscape and align with a previous OGM‐based study reporting additional findings in ~30% of CN‐AML cases (CN‐LOH excluded) [22].

In our cohort, the presence of an alteration was also associated with worse survival. Many of the alterations identified in this study, such as *RUNX1* disruptions, *NUP98::NSD1, KMT2A*‐PTD, *NF1* deletions and CN‐LOH involving mutated leukemia genes, have previously been associated with adverse prognosis in AML. This could provide a partial biological explanation for the observed association with survival. In addition, OGM positive cases were more likely to belong in the non‐favorable ELN2022 risk groups and were less likely to be *NPM1*‐mutated. This is likely to contribute to the observed association with poor prognosis and is in line with previous studies indicating that *NPM1* mutated AML typically carries a relatively low burden of CNVs [23, 24]. However, OGM‐positive cases within the favorable ELN risk category also showed poor survival and may represent an interesting subgroup with an unfavorable prognosis despite their favorable classification, warranting further studies. Furthermore, within the *NPM1* mutated cases, OGM findings were significantly associated with worse survival, in line with a recent study showing that additional co‐occurring molecular mutations can markedly alter the prognosis of *NPM1* mutated AML [25]. Our results suggest that SVs may also have similar effects. Although the cohort size and lack of stratification by treatment protocols limit the strength of prognostic conclusions, our findings indicate that comprehensive SV analysis by OGM could contribute to improving risk‐stratification of CN‐AML. Larger studies will be required to confirm these associations and to determine which specific SVs or genes most strongly drive it.

Recurrent alterations reported in earlier OGM analyses of AML, including CN‐AML cases [22], such as *KMT2A*‐PTD, *NUP98*::*NSD1* and *TET2* deletions, were also identified in our study. In addition to these, *NF1* deletions, *RUNX1* disruptions, and 13q14 (*DLEU*/*RB1*) deletions were identified as recurrent events in our CN‐AML cohort. Among individual gene‐level findings, *RUNX1* disruptions are well‐established adverse‐risk events in the ELN classification, whereas accumulating evidence suggests that *NUP98* rearrangements should also be considered adverse‐risk abnormalities [5, 26]. *NUP98*::*NSD1* fusion has been reported to occur in 2% of CN‐AML cases and is associated with poor outcomes and limited response to standard therapies [27]. Our results support previous studies demonstrating that OGM provides an efficient method for genome‐wide detection of cryptic balanced translocations, such as those involving *NUP98* [16, 28]. Regarding *RUNX1*, our findings highlight the value of OGM for detecting clinically impactful gene disruptions caused by smaller SVs (both deletions and duplications). Based on the VAFs, we could not rule out the possibility of a germline *RUNX1* alteration in each case without confirmation from other tissues. However, to our knowledge, none of these alterations have been reported as germline or founder mutations in the Finnish population. *KMT2A‐*PTD was the most frequent alteration in our cohort, occurring in 10% of cases, consistent with published data [29]. Although not recognized as a distinct genetic subgroup in WHO/ELN, recent studies indicate that *KMT2A*‐PTD, particularly in CN‐AML, may confer adverse prognosis [30, 31, 32]. Importantly, *KMT2A‐*PTD is not detectable with karyotyping and FISH and can also be challenging to detect by standard NGS assays, especially when present at low VAF. Our results are consistent with previous studies demonstrating that OGM is a reliable method for identifying *KMT2A*‐PTD [33, 34, 35].

Our dataset also contained recurrent deletions of *NF1* and the 13q14 (*DLEU‐*region). Deletions at 13q14 are frequent in CLL [36] but rare in AML, making their recurrence in our CN‐AML cohort unexpected. One of the 13q14 deletions arose from a simple deletion, whereas the second resulted from translocations between chromosomes 11 and 13 that led to simultaneous deletions of *ATM* on chromosome 11 and *DLEU*‐region and *RB1* on chromosome 13. Curiously, deletions at both of these loci (*ATM* and *DLEU*) are typical abnormalities in CLL [36], but co‐occurring deletions of these loci are, to our knowledge, very rare in AML. *NF1* deletions, although infrequent in AML, can contribute to leukemogenesis by impairing RAS pathway regulation [37]. Their detection in two patients, one of whom had likely biallelic *NF1* inactivation (deletion and a frameshift mutation), underscores the value of OGM for identifying cryptic tumor suppressor gene losses. Other identified deletions involved *TET2, FLT3* and *PRPF8*, all which have known roles in myeloid malignancies. The *TET2* deletion co‐occurred with a high‐VAF stop‐gain mutation, supporting a biallelic loss‐of‐function mechanism reported for *TET2* [38]. The *FLT3* deletion was estimated to encompass exons 1–9 and co‐occurred with an *FLT3*‐ITD, presumably on the other allele. This configuration is consistent with a high *FLT3*‐ITD allelic ratio, associated with poor prognosis [39], and illustrates how OGM can clarify multi‐hit gene states in clinically relevant genes. *PRPF8* deletions are rarely reported in AML, but they have been implicated in RNA splicing defects contributing to leukemogenesis [40].

Large CN‐LOH events entailing telomeric regions were identified in 15% of cases, most frequently affecting chromosome 13q, consistent with previous reports [41]. CN‐LOH can play a key role by duplicating mutant allele, while eliminating the corresponding wild‐type allele [42]. Accordingly, four CN‐LOH cases in our cohort harbored high‐VAF mutations in leukemia genes, including *WT1*, *RUNX1*, *FLT3* and *TET2*. While CN‐LOH events are not routinely assessed in AML despite their potential clinical relevance [41, 43], our approach using OGM label‐SNP co‐occurrence strengthens confidence in the CN‐LOH calls made by OGM and illustrates that OGM can provide allele‐state information in copy‐neutral context. However, it should be noted that systematic evaluations of OGM performance in CN‐LOH detection as well as the added value of incorporating 7000–8000 SNP markers are lacking and beyond the scope of this study. In addition, CN‐LOH detection by OGM still requires high tumor cell content.

Our dataset also included SVs that did not overlap with the leukemia‐associated gene list used. Although no recurrent events were identified in the cohort, one notable finding was a balanced translocation t(3;20)(p13;q13.12) involving *FOXP1* and *EYA2*. The gene and rearrangement orientations could allow the formation of a fusion gene, but due to lack of suitable material we were not able to confirm this at RNA level. However, *FOXP1* is a transcription factor that has been implicated in chemotherapy resistance in AML [44] and reported as a part of another fusion (*FOXP1::PDGFRA*) in myeloproliferative neoplasm with eosinophilia [45]. Our finding may therefore represent a rare, novel *FOXP1*‐involving fusion. Altogether, larger cohorts will be required to determine whether some of the identified novel SVs, such as *PUM3* deletion or *FOXP1* rearrangements, represent recurrent events in AML. In this context, CN‐AML may offer a unique opportunity to identify biologically relevant alterations, as these cases lack the extensive genomic instability seen in more complex karyotypes; therefore, when a novel SV is present in this relatively stable genomic background, it could be more likely to represent a functionally meaningful driver rather than a passenger event.

In conclusion, our results confirm that OGM is a powerful genome‐wide method capable of detecting a broad range of novel and clinically relevant SVs in CN‐AML. Notably, nearly half of the CN‐AML cases in our cohort harbored large alterations or SVs involving leukemia‐associated genes. The growing number of newly described SVs and fusions in leukemias, including AML [46, 47, 48], underscores the need for diagnostic approaches capable of whole‐genome SV interrogation. As SV datasets expand and become linked to clinical and molecular data, they can provide a foundation for more refined future risk models [49], and may also facilitate the discovery of novel genes and pathways involved in AML pathogenesis.

## Author Contributions

**Tuuni Turtinen:** methodology, visualization, investigation, writing – original draft, writing – review and editing. **Andriana Valkama:** investigation, writing – original draft, methodology, visualization, writing – review and editing. **Christopher Wray:** investigation, methodology, writing – review and editing, visualization. **Sandra Vorimo:** writing – review and editing, methodology. **Hannele Räsänen:** writing – review and editing. **Eeva‐Riitta Savolainen:** supervision, writing – review and editing. **Katri Pylkäs:** project administration, writing – review and editing, supervision. **Tuomo Mantere:** conceptualization, investigation, methodology, funding acquisition, writing – original draft, project administration, supervision, writing – review and editing, visualization.

## Funding

This work was supported by the Sigrid Juselius Foundation (grant number 220111) and the Academy of Finland (grant number 360442).

## Ethics Statement

The study was approved by the Institutional Ethical Board of the North Ostrobothnia Health Care District (52/2021) and the Scientific Advisory Board of the Finnish Hematology Registry and Clinical Biobank. Informed consent was obtained from all subjects as part of the biobank collection.

## Conflicts of Interest

The authors declare no conflicts of interest.

## Supporting information


**Table S1:** Patient characteristics, genetic variant information (OGM and NGS), and the ELN, FAB and WHO classifications for the CN‐AML cohort.
**Table S2:** Genes targeted in panel sequencing, minimum average read depth, and sequencing technology used.
**Table S3:** List of leukemia‐associated genes and their coordinates used in the OGM analysis (hg38).
**Table S4:** RNAseq quality metrics.
**Table S5:** Technical quality metrics of the analyzed CN‐AML samples.
**Table S6:** Ten most differentially up‐ and downregulated genes in *KMT2A*‐PTD cases.
**Table S7:** Somatic SVs that do not overlapwith known leukemia‐associated genes.
**Table S8:** Results from SNP label‐site overlap CN‐LOH analysis.

**Figure S1:** *KMT2A* partial tandem duplications identified by OGM in five samples (A–E). Each reference genome map (green) is aligned to the KMT2A illustration on top and tandem repeats presented in orange bars in each sample assembly (blue).
**Figure S2:** *RUNX1* disruptions detected by OGM. (A) 21q22.12 deletion of *RUNX1* exons 1 and 2 (2.5 kbp). (B) 21q22.12q22.13 deletion of *RUNX1* (1.9 Mbp). (C) 21q22.12 intragenic duplication of *RUNX1* exons 3 to 6. Duplicated area presented in orange bars in sample assembly.
**Figure S3:** Deletions. (A) 13q14.2q14.3 deletion of *DLEU*‐region (1.5 Mbp). (B) 17q11.2 deletion of *NF1* (1.3 Mbp). (C) 17q11.2 deletion (50 kbp) of *NF1* entailing multiple exons. (D) 4q24 deletion of *TET2* (430 kbp). (E) 17p13.3p13.1 deletion of *PRPF8* (5.6 Mbp) marked with red arrows. (F) 13q12.2q14.11 deletion of *FLT3* exons 1–9 (12.8 Mbp) marked with red arrows.
**Figure S4:** Complex rearrangements. (A) Inter‐ and intrachromosomal rearrangements affecting 11p11.2q23.1 and 13q14.2q21.2 and resulting in deletions of *ATM*, *RB1* and the *DLEU*‐region. Deleted areas indicated in red rectangles. (B) Multiple focal SVs in 9q34 region entailing *ABL1* and *NUP214*.
**Figure S5:** Balanced translocations. (A) A known t(5;11)(q35.3; p15.4) leading to *NUP98::NSD1* fusion gene, and (B) a putative novel fusion between *FOXP1* and *EYA2* due to balanced translocation t(3;20)(p13;q13.12).

## Data Availability

The RNA sequencing raw data generated in this study have been returned to the Hematological Biobank and can be obtained upon approval by the biobank's steering committee (for details on the process, see www.hematologinenbiopankki.fi or contact hembio@helsinki.fi). Diagnostic NGS results for this study were obtained from the Hematological Biobank and can be accessed similarly upon approval by the biobank's steering committee. The other data that support the findings of this study are available from the corresponding author on reasonable request.
