# Supplementary material for: Optical Genome Mapping Reveals Frequent Cryptic Structural Aberrations in Normal Karyotype Acute Myeloid Leukemia
Source: Int J Cancer. 2026 May 14;159(5):1218–27. doi: 10.1002/ijc.70548 (PMC13340983; doi:10.1002/ijc.70548)
Supplement: Supplementary file 2 — Figure S1: KMT2A partial tandem duplications identified by OGM in five samples (A–E). Each reference genome map (green) is aligned to the KMT2A illustration on top and tandem repeats presented in orange bars in each sample assembly (blue). Figure S2: RUNX1 disruptions detected by OGM. (A) 21q22.12 deletion of RUNX1 exons 1 and 2 (2.5 kbp). (B) 21q22.12q22.13 deletion of RUNX1 (1.9 Mbp). (C) 21q22.12 intragenic duplication of RUNX1 exons 3 to 6. Duplicated area presented in orange bars in sample assembly. Figure S3: Deletions. (A) 13q14.2q14.3 deletion of DLEU‐region (1.5 Mbp). (B) 17q11.2 deletion of NF1 (1.3 Mbp). (C) 17q11.2 deletion (50 kbp) of NF1 entailing multiple exons. (D) 4q24 deletion of TET2 (430 kbp). (E) 17p13.3p13.1 deletion of PRPF8 (5.6 Mbp) marked with red arrows. (F) 13q12.2q14.11 deletion of FLT3 exons 1–9 (12.8 Mbp) marked with red arrows. Figure S4: Complex rearrangements. (A) Inter‐ and intrachromosomal rearrangements affecting 11p11.2q23.1 and 13q14.2q21.2 and resulting in deletions of ATM, RB1 and the DLEU‐region. Deleted areas indicated in red rectangles. (B) Multiple focal SVs in 9q34 region entailing ABL1 and NUP214. Figure S5: Balanced translocations. (A) A known t(5;11)(q35.3; p15.4) leading to NUP98::NSD1 fusion gene, and (B) a putative novel fusion between FOXP1 and EYA2 due to balanced translocation t(3;20)(p13;q13.12). [file IJC-159-1218-s001.pdf]

Optical genome mapping reveals frequent cryptic structural aberrations in normal karyotype acute myeloid leukemia

Tuuni Turtinen, Andriana Valkama, Christopher Wray, Sandra Vorimo, Hannele Räsänen, Eeva-Riitta Savolainen, Katri Pylkäs & Tuomo Mantere

Table of contents

Supplementary figures

Figure S1 (A)–(E). *KMT2A* partial tandem duplications 1

Figure S2 (A)–(C). *RUNX1* disruptions 2

Figure S3 (A)–(F). Deletions 3

Figure S4 (A)–(B). Complex rearrangements 4

Figure S5 (A)–(B). Translocations 5

Supplementary tables (available in separate Excel file)

Table S1. Patient characteristics, genetic variant information (OGM and NGS), and the ELN, FAB and WHO classifications for the CN-AML cohort

Table S2. Genes targeted in panel sequencing, minimum average read depth, and sequencing technology used

Table S3. List of leukemia-associated genes and their coordinates used in the OGM analysis (hg38)

Table S4. RNAseq quality metrics

Table S5. Technical quality metrics of the analyzed CN-AML samples

Table S6. 10 most differentially up- and downregulated genes in *KMT2A*-PTD cases

Table S7. Somatic SVs that do not overlap with known leukemia-associated genes

Table S8. Results from SNP label-site overlap CN-LOH analysis

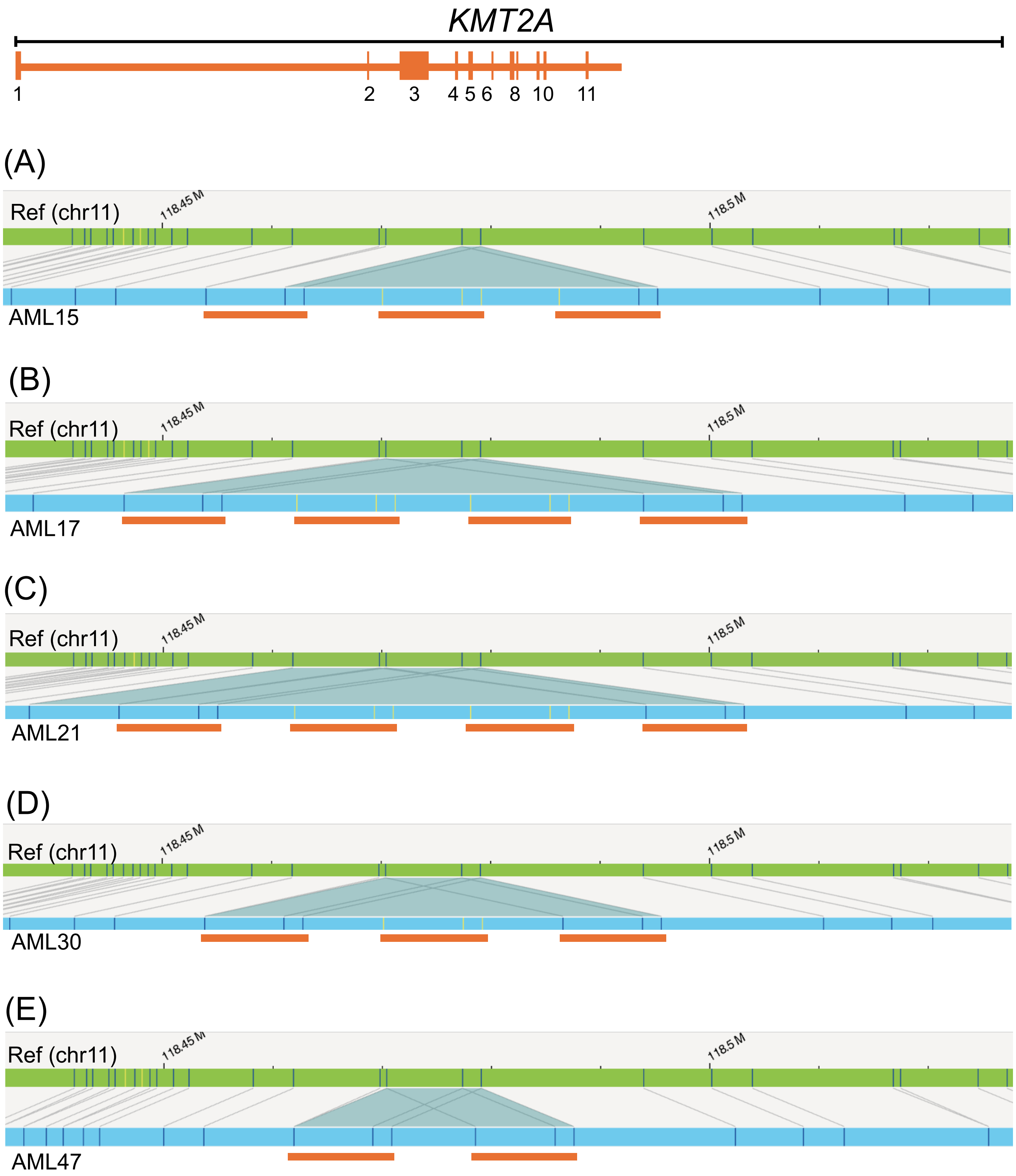

Figure S1. *KMT2A* partial tandem duplications identified by OGM in five samples (A)–(E). Each reference genome map (green) is aligned to the *KMT2A* illustration on top and tandem repeats presented in orange bars in each sample assembly (blue).

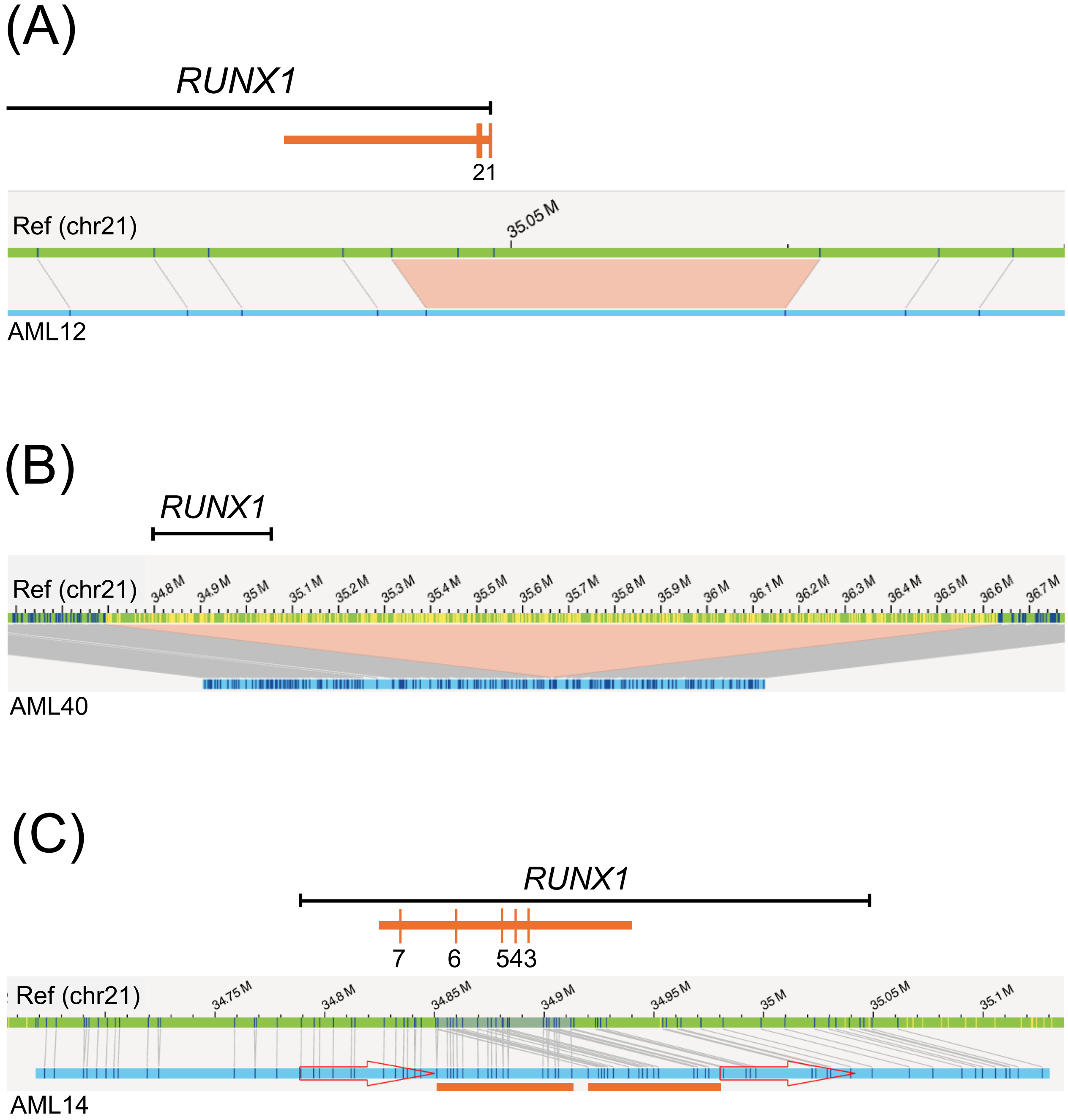

Figure S2. *RUNX1* disruptions detected by OGM. (A) 21q22.12 deletion of *RUNX1* exons 1 and 2 (2.5 kbp). (B) 21q22.12q22.13 deletion of *RUNX1* (1.9 Mbp). (C) 21q22.12 intragenic duplication of *RUNX1* exons 3 to 6. Duplicated area presented in orange bars in sample assembly.

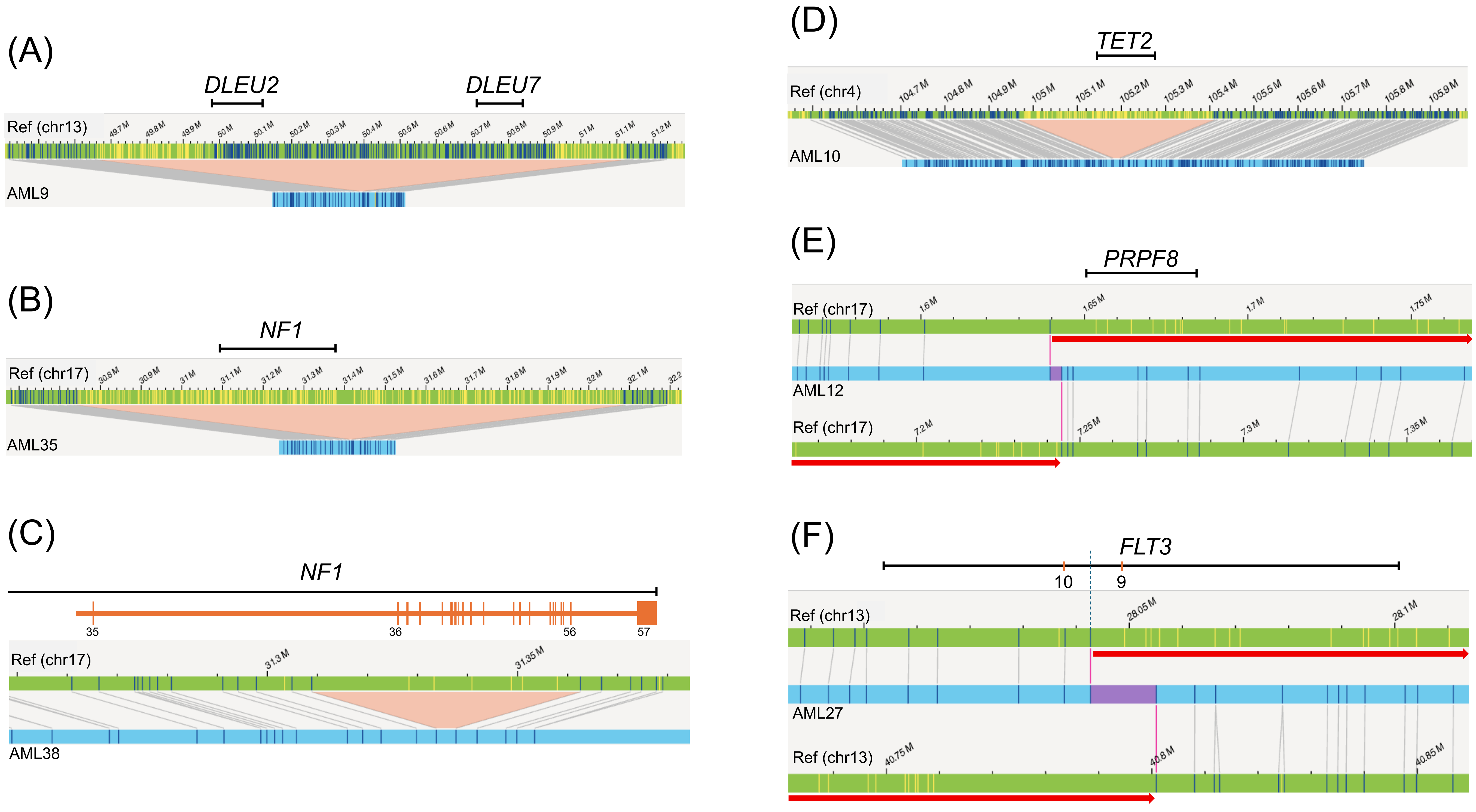

Figure S3. Deletions. (A) 13q14.2q14.3 deletion of *DLEU*-region (1.5 Mbp). (B) 17q11.2 deletion of *NF1* (1.3 Mbp). (C) 17q11.2 deletion (50 kbp) of *NF1* entailing multiple exons. (D) 4q24 deletion of *TET2* (430 kbp). (E) 17p13.3p13.1 deletion of *PRPF8* (5.6 Mbp) marked with red arrows. (F) 13q12.2q14.11 deletion of *FLT3* exons 1-9 (12.8 Mbp) marked with red arrows.

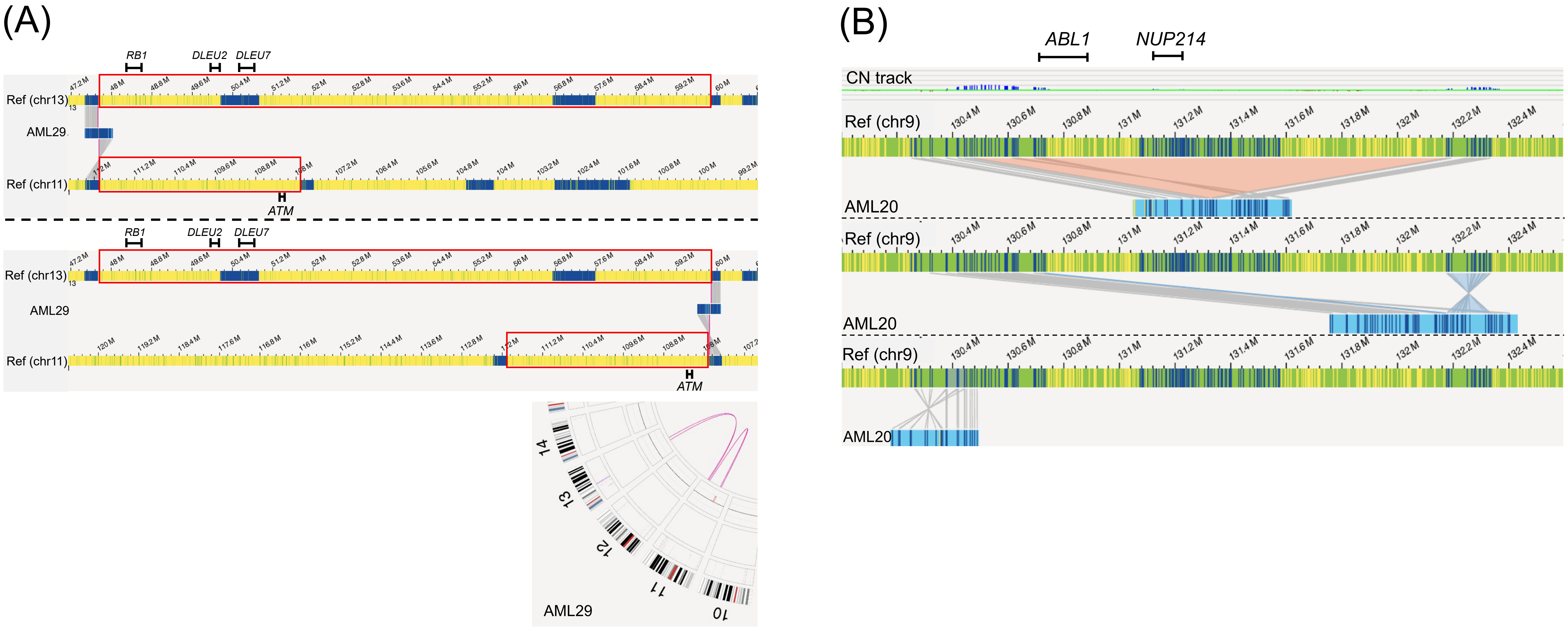

Figure S4. Complex rearrangements. (A) Inter- and intrachromosomal rearrangements affecting 11p11.2q23.1 and 13q14.2q21.2 and resulting in deletions of *ATM, RB1* and the *DLEU*-region. Deleted areas indicated in red rectangles. (B) Multiple focal SVs in 9q34 region entailing *ABL1* and *NUP214*.

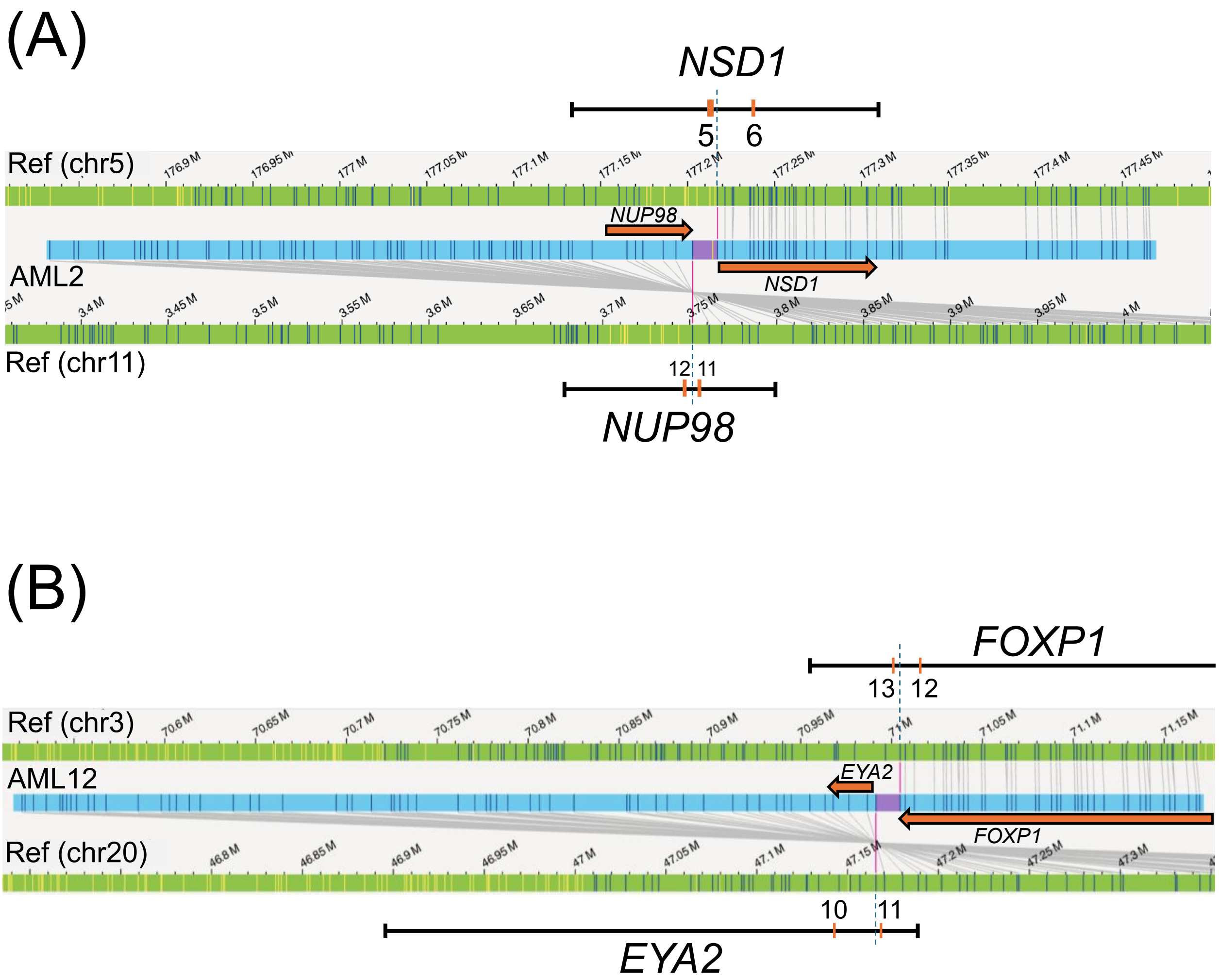

Figure S5. Balanced translocations. (A) A known t(5;11)(q35.3;p15.4) leading to *NUP98::NSD1* fusion gene, and (B) a putative novel fusion between *FOXP1* and *EYA2* due to balanced translocation t(3;20)(p13;q13.12).
